# The Complementarity Principle—One More Step towards Analytical Docking on the Example of Dihydrofolate Reductase Complexes

**DOI:** 10.3390/life11090983

**Published:** 2021-09-19

**Authors:** Vladimir Potemkin, Maria Grishina

**Affiliations:** Laboratory of Computational Modelling of Drugs, South Ural State University, 454080 Chelyabinsk, Russia

**Keywords:** 3D maps of electron density, complementarity, dihydrofolate reductase, docking

## Abstract

New approaches to assessing the “enzyme–ligand” complementarity, taking into account hydrogens, have been proposed. The approaches are based on the calculation of three-dimensional maps of the electron density of the receptor–ligand complexes. The action of complementarity factors, first proposed in this article, has been demonstrated on complexes of human dihydrofolate reductase (DHFR) with ligands. We found that high complementarity is ensured by the formation of the most effective intermolecular contacts, which are provided due to predominantly paired atomic–atomic interactions, while interactions of the bifurcate and more disoriented type are minimized. An analytical docking algorithm based on the proposed receptor–ligand complementarity factors is proposed.

## 1. Introduction

At present, considerable experimental material has been accumulated on the chemical transformations of organic compounds. The presence of certain functional groups in the substrate allows chemists to assume the types of reactions, reagents, and conditions for carrying out the reactions to achieve the desired product. Nevertheless, the task of targeted synthesis is often complicated by the presence in the substrate and reagent of several functional groups capable of interacting with each other, the possibility of competing parallel reactions. Questions often arise as to which of them will prevail and how the process can be controlled to obtain the required product. A special case of interactions is enzyme–ligand complexation. The development of X-ray structural analysis of complexes makes it possible to freeze the structures of the complexes at the moment of interaction between the receptor and the ligand. Various docking techniques are being developed to model the structures of enzyme–ligand complexes but, nevertheless, they often give complexes that are very different from those found experimentally. Different scoring functions and binding energies do not allow the selection of the most correct structures of the complexes. Therefore, in this work, we tried to take a different path. We assumed that the interaction between the enzyme and the ligand, as a particular case of substrate–reagent interactions, obeys a certain law of complementarity to their electronic structures which dictates a completely definite orientation of the ligand in the receptor cavity.

Here we must point out that the idea of complementarity is not new. It was first formulated by Paul Ehrlich in biology for antigen–antibody interactions [1]. In physics, the idea of complementarity of physical properties was formulated by Niels Bohr [2] and presented in the form of Heisenberg’s uncertainty principle [3,4,5]. Even later, Watson and Crick published data on the complementarity of nucleotide pairs in DNA and RNA molecules [6]. We tried to develop the idea of complementarity in chemical systems to present it in the form of a mathematical expression relating the electron densities of a receptor and a ligand in their complexes.

Recently, we investigated [7,8,9] different enzyme–ligand complexes (namely, complexes of cyclin-dependent protein kinases, mouse acetylcholinesterase, HIV-1 protease, EGFR) taken from the Protein Data Bank [10,11]. Three-dimensional maps of electron density were constructed for the complexes using the AlteQ method [12,13,14,15,16]. We found the complementarity factor which describes enzyme–ligand complementarity of the complexes without hydrogens very well [7,8,9]. It was shown that this method can be used for the search for the most correct binding poses of ligands during docking procedures [9]. In the current paper, for the first time, a detailed assessment of the parameters of the electronic structure of enzyme–ligand complexes, taking into account hydrogen atoms, is carried out, the complementarity method was improved for these aims, and new complementarity factors were proposed.

Dihydrofolate reductase (DHFR) is one of the major enzymes determining intracellular metabolism. Briefly, under a cofactor nicotinamide adenine dinucleotide phosphate (NADPH) regulation, DHFR transforms folic acid taken from food to 7,8-dihydrofolate and 5,6,7,8-tetrahydrofolate (FH4) [17,18]. At that, FH4 is a significant cofactor for the enzymes determining the transfer of carbon groups and influencing synthesis of different amino acids and purines. Thus, the lack of FH4 disrupts this process, resulting in DNA degradation with subsequent cell death [19,20,21]. Therefore, DHFR is a very important target for chemotherapy [22]. Thus, the investigation of interactions of ligands with DHFR can be useful in the rational drug design.

## 2. Materials and Methods

### 2.1. Considered Complexes

For the complementarity assessment, complexes of human DHFR with ligands were taken from the Protein Data Bank [10,11]. These are 1boz, 1hfp, 1kms, 3ghc, 3gi2, 3ntz, 3nu0, 4kfj, 4qhv [23,24,25,26,27,28,29]. We evaluated the enzyme–ligand complementarity for human DHFR complexes without hydrogen atoms in the same way as it was performed in [7,8,9] for complexes of other enzymes.

Then, we performed detailed enzyme–ligand complementarity assessment for the complexes with added hydrogen atoms. At that, hydrogens positions were optimized using hybrid QM/MM approach. The used optimization technique and electronic properties of the complexes have been described in [30].

### 2.2. D Maps of Electron Density

To estimate the electron density, we used the AlteQ method, which has proven itself well in predicting the electron density maps of a number of organic and inorganic compounds and determined using low temperature high resolution X-ray diffraction data [12,13,14,15,16].

In the AlteQ method, the electron density (*ρ*) at an arbitrary *m*th point in the molecular space with coordinates $x_{m},y_{m},z_{m}$ can be represented as follows (Equations (1) and (2)):

(1) $$\rho \left(x_{m},y_{m},z_{m}\right)=\sum_{A=1}^{N}\rho_{Am\left(all\;shells\right)},$$

(2) $$\rho_{Am\left(all\;shells\right)}=\overset{n_{A}}{\underset{i=1}{\sum }}a_{A_{isp}}\exp\left(-b_{A_{isp}}R_{Am}\right)++\overset{n_{A}-1}{\underset{i=3}{\sum }}a_{A_{id}}\exp\left(-b_{A_{id}}R_{Am}\right)+\overset{n_{A}-2}{\underset{i=4}{\sum }}a_{A_{if}}\exp\left(-b_{A_{if}}R_{Am}\right)$$

where *N* is the number of atoms in a molecule and $\rho_{Am}$ is the *A* atomic increment in molecular electron density at the *m*th point of the molecular space, $a_{A_{isp}}$, $b_{A_{isp}}$, $a_{A_{id}}$, $b_{A_{id}}$, $a_{A_{if}}$, $b_{A_{if}}$ are AlteQ atomic parameters describing the *i*-th sp-orbital, d-orbital, and the f-orbital of the *A* atom respectively, $n_{A}$ is the period number of the *A* atom, $R_{Am}$ is the distance between the *A* atom and the *m*th point. If $R_{Am}$ is measured in Å, then the units of the AlteQ coefficients are [*b*] = 1/Å, [*a*] = e/Å^3^, and consequently [*ρ*] = e/Å^3^.

Therefore, we can estimate the electron density contributions of the ligand and the enzyme to the *m*th point of the molecular space (Equations (3) and (4)):

(3) $$\rho_{L\left(all\;shells\right)}^{}\left(x_{m},y_{m},z_{m}\right)=\sum_{\begin{array}{c} A=1\\ A\in ligand \end{array}}^{N_{ligand}}\rho_{Am\left(all\;shells\right)}\;,$$

(4) $$\rho_{E\left(all\;shells\right)}^{}\left(x_{m},y_{m},z_{m}\right)=\overset{N_{enzyme}}{\underset{\begin{array}{c} A=1\\ A\in enzyme \end{array}}{\sum }}\rho_{Am\left(all\;shells\right)}\;$$

where $N_{enzyme}$ and $N_{ligand}$ are the numbers of atoms of the ligand and the enzyme.

Three-dimensional maps of electron density represented as the density value at the lattice junctions of the cubic grid with the step 0.1 Å have been calculated for all complexes. All atoms (atoms of water molecules, chloride anions, sulfate anions, etc., included in the experimental complexes) not belonging to the ligand molecule were referred to the enzymatic part (enzyme).

It is obvious that the outer shell plays the most important role in the formation of covalent bonds and intermolecular contacts, therefore, first of all, we obtained the contribution of the outer shells ($\rho_{Am\left(outer\right)}$) as follows:

(5) $$\rho_{Am\left(outer\;shells\right)}=a_{A_{nsp}}\exp\left(-b_{A_{nsp}}R_{Am}\right)+a_{A_{\left(n-1\right)d}}\exp\left(-b_{A_{\left(n-1\right)d}}R_{Am}\right)+a_{A_{\left(n-2\right)f}}\exp\left(-b_{A_{\left(n-2\right)f}}R_{Am}\right)\;,$$

For s- and *p*-elements of the 1–3 periods (ligand atoms and near-ligand enzyme atoms are mostly s- and *p*-elements of the 1–3 periods), the contribution of the outer orbital of the *A* atom to the electron density at the *m*th point can be written as follows:

(6) $$\rho_{Am\left(outer\right)}=a_{A_{n_{A}sp}}\exp\left(-b_{A_{n_{A}sp}}R_{Am}\right),$$

The contribution of the inner shells ($\rho_{Am\left(inner\right)}$) has been estimated as follows (Equation (7)):

(7) $$\rho_{Am\left(inner\right)}=\rho_{Am\left(all\;shells\right)}-\rho_{Am\left(outer\right)},$$

Therefore, analogously to the Equations (5) and (6) we can estimate the inner shells ($\rho_{L\left(inner\right)}^{}$, $\rho_{E\left(inner\right)}^{}$) and the outer shells ($\rho_{L}^{}$, $\rho_{E}^{}$) electron density contributions of the ligand and the enzyme to the *m*th point of the molecular space, e.g., (Equations (8)–(11)):

(8) $$\rho_{L\left(inner\right)}^{}=\sum_{\begin{array}{c} A=1\\ A\in ligand \end{array}}^{N_{ligand}}\rho_{Am\left(inner\right)}\;,$$

(9) $$\rho_{E\left(inner\right)}^{}=\sum_{\begin{array}{c} A=1\\ A\in enzyme \end{array}}^{N_{enzyme}}\rho_{Am\left(inner\right)}\;,$$

(10) $$\rho_{L}^{}=\sum_{\begin{array}{c} A=1\\ A\in ligand \end{array}}^{N_{ligand}}\rho_{Am\left(outer\right)}\;,$$

(11) $$\rho_{E}^{}=\sum_{\begin{array}{c} A=1\\ A\in enzyme \end{array}}^{N_{enzyme}}\rho_{Am\left(outer\right)}\;,$$

First, detailed 3D electron density maps were calculated for the entire near-ligand space, where the electron density of the ligand is greater than 0.001 au (namely, at the *m*th points of the molecular space with $\rho_{L}>0.001$ e/Bohr^3^). In other words, this space is the set of traditional ligand atomic basins—the term which is used in ‘Theory of Atoms in Molecules’ suggested by Richard F.W. Bader [31,32]. Let us call this zone the ligand zone.

Second, 3D maps of electron density were evaluated in the zones of the maximum enzyme–ligand overlap, namely in the space where the electron densities of the enzyme and the ligand are greater than 0.001 au (namely, at *m* points of molecular space with $\rho_{E}>0.001$ au and $\rho_{L}>0.001$ au) in the same way as it was performed in [7,8,9] for other complexes. Let us call these zones the zones of intermolecular contacts.

### 2.3. Complementarity Factors

An attempt to find a mathematical relationship between the electron density of the enzyme and the ligand showed that there are good correlations of complementarity factors ($CF_{k}$) with the distance descriptor SUMRLRE:

(12) $$CF_{k}=a_{CFk}+b_{CFk}\cdot SUMRLRE,$$

(13) $$SUMRLRE=R_{ml}+R_{me}$$

where $R_{ml}$ is the distance between the *m-*th point in space and the *l*-th ligand’s atom with the greatest contribution to the ligand’s electron density at that point. $R_{me}$ is, analogously, the distance between the *m-*th point and *e*-th enzyme’s atom with the greatest contribution to the enzyme’s electron density at that point. $a_{CFk}$ and $b_{CFk}$ are parameters of the Equation (12) dependent on the enzyme–ligand complex and the type *k* of the complementarity factor.

The first complementarity factor (CF1) has been suggested in [7,8,9] and it equals:

(14) $$CF1=ln\left(\frac{\rho_{E}\cdot \rho_{e\left(CNT\right)}}{N_{e}}\right)+ln\left(\frac{\rho_{L}\cdot \rho_{l\left(CNT\right)}}{N_{l}}\right),$$

where $\rho_{E}$ and $\rho_{L}$ are the outer shells electron densities of the enzyme and the ligand at the *m-*th point (in e/Å^3^), $\rho_{e\left(CNT\right)}$ and $\rho_{l\left(CNT\right)}$ are the electron densities at the centers of the *e-*th enzyme atom and the *l-*th ligand atom, respectively, $N_{e}$ and $N_{l}$ are the atomic numbers of the *e-*th enzyme atom and the *l-*th ligand atom, respectively.

The second (CF2) and the third (CF3) complementarity factors equal:

(15) $$CF2=ln\left(\frac{\rho_{E}\cdot \rho_{e\left(CNT\right)}}{N_{e\left(outer\right)}^{}}\right)+ln\left(\frac{\rho_{L}\cdot \rho_{l\left(CNT\right)}}{N_{l\left(outer\right)}^{}}\right),$$

(16) $$CF3=ln\left(\frac{\rho_{E}\cdot \rho_{e\left(CNT\right)}}{N_{e\left(outer\right)}^{2}}\right)+ln\left(\frac{\rho_{L}\cdot \rho_{l\left(CNT\right)}}{N_{l\left(outer\right)}^{2}}\right)$$

where $N_{e\left(outer\right)}$ and $N_{l\left(outer\right)}$ are the numbers of the outer electrons of the *e-*th enzyme atom and the *l-*th ligand atom, respectively.

It is obvious that the intermolecular interaction between molecules is such until a covalent bond arises, which is characterized by shorter contacts while the probability of overlapping of the inner layers increases. Therefore, to assess the degree of complementarity of molecules for intermolecular interactions, it is necessary to take into account the overlaps of the inner layers in the intermolecular contact zone; such overlaps should tend to zero for non-covalently bound complexes. To take into account the possibility of the formation of an incorrect complex in the docking procedures, we propose to use the complementarity factor CF4:

(17) $$CF4=ln\left(\frac{\rho_{E}\cdot \rho_{e\left(CNT\right)}}{N_{e\left(outer\right)}^{2}}\right)+ln\left(\frac{\rho_{L}\cdot \rho_{l\left(CNT\right)}}{N_{l\left(outer\right)}^{2}}\right)+\rho_{E\left(inner\right)}+\rho_{L\left(inner\right)},$$

where $\rho_{E\left(inner\right)}$ and $\rho_{L\left(inner\right)}$ are the electron densities of the inner shells of the enzyme and ligand at the *m*th point.

### 2.4. Overlaps

Different kinds of enzyme–ligand overlaps have been computed using the integration over atomic basins described in detail in [15,33]. The number of electrons in the “enzyme inner orbital–ligand inner orbital” overlaps of the complexes equals (Equation (18)):

(18) $$n_{L\left(inner\right)\cap E\left(inner\right)}=\int_{\sigma_{L}}^{}\rho_{L\left(inner\right)\cap E\left(inner\right)}dV,$$

where $\sigma_{L}$ is the set of ligand atomic basins (the ligand zone), dV is the differential of the volume. The overlap electron density function ($\rho_{L\left(inner\right)\cap E\left(inner\right)}$) depends on the $\rho_{L\left(inner\right)}$ and $\rho_{E\left(inner\right)}$ electron density functions of the ligand and enzyme, respectively.

The number of electrons in the overlaps of the inner and outer orbitals of the enzyme and the ligand equals (Equation (19)):

(19) $$n_{\left(outer\right)\cap \left(inner\right)}=\overset{}{\underset{\sigma_{L}}{\int }}\rho_{inner\cap outer}dV==\overset{}{\underset{\sigma_{L}}{\int }}\rho_{L\left(inner\right)\cap E}dV+\overset{}{\underset{\sigma_{L}}{\int }}\rho_{L\cap E\left(inner\right)}dV=n_{L\left(inner\right)\cap E\left(outer\right)}+n_{L\left(outer\right)\cap E\left(inner\right)}$$

$n_{\left(outer\right)\cap \left(inner\right)}$ is determined by the overlap electron density function of the inner and outer orbitals of the enzyme and the ligand ($\rho_{inner\cap outer}=\rho_{L\left(inner\right)\cap E}+\rho_{L\cap E\left(inner\right)}$) which in their turn depend on the $\rho_{L\left(inner\right)}$, $\rho_{E}$, $\rho_{L}$ and $\rho_{E\left(inner\right)}$ electron density functions of the ligand and enzyme.

The number of electrons in the overlaps of the outer orbitals of the enzyme and the ligand equals (Equation (20)):

(20) $$n_{L\cap E}=\int_{\begin{array}{c} zones\;of\\ \begin{array}{c} intermolecular\\ contacts \end{array} \end{array}}^{}\rho_{L\cap E}dV,$$

where $\rho_{L\cap E}$ is the overlap electron density function of the outer orbitals of the enzyme and the ligand.

The algorithm for determining overlap functions whose particular cases are $\rho_{L\left(inner\right)\cap E\left(inner\right)}$, $\rho_{L\left(inner\right)\cap E}$, $\rho_{L\cap E\left(inner\right)}$, $\rho_{L\cap E}$) is described in [33].

## 3. Results and Discussion

### 3.1. Complexes without Hydrogens

We estimated the “enzyme–ligand” overlaps in the complexes without hydrogens. It has been found that the overlaps $n_{L\cap E}$ of non-hydrogen atoms are significant and determine complexation. Most of them are overlaps of electronegative atoms of NH2, COOH, OH groups forming hydrogen bonds. In this case, the hydrogen atoms are partially transparent, allowing the electron density of the enzyme and ligand’s electronegative atoms to pass through hydrogens for additional overlaps. The number of electrons in the “enzyme–ligand” overlaps ($n_{L\cap E}$) and the number of electrons donated by the ligand ($n_{L\cap E}^{ligand}$) to the overlaps are shown in Table 1. At that, we obtained that the ligands donate the larger number of electrons to the overlaps than the enzyme.

All complexes showed very high complementarity of ligands and the enzyme structures without hydrogens to each other in the zones of intermolecular contacts (with $\rho_{E}>0.001$ au and $\rho_{L}>0.001$ au). Recently published CF1 factor correlates with SUMRLRE descriptor very well. Squared correlation coefficients of the dependencies (12) (R2(CF1)) vary within 0.872–0.951. Maximum value of the CF1 factor (MAX(CF1)) ranges within −2.653–−4.548 demonstrating highly efficient contacts in the complexes.

**Figure 1:**
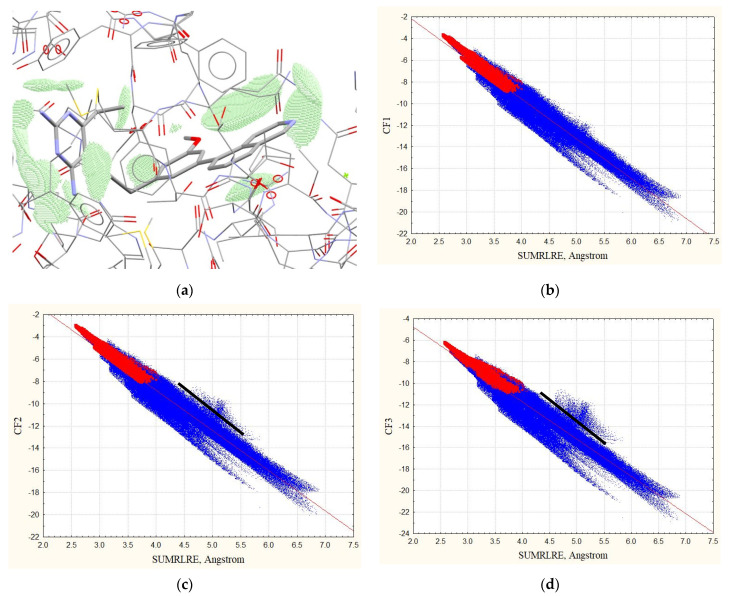
(**a**) Zones of intermolecular contacts of the ligand of 4kfj complex without hydrogens (with $\rho_{E}>0.001$ au and $\rho_{L}>0.001$ au). The dependencies of the complementarity factors with SUMRLRE on the example of 4kfj complex: (**b**) *CF1*, (**c**) *CF2*, (**d**) *CF3*. Red points show the dependencies for the zones of intermolecular contacts (the number of red points N_points_ = 20,129), the total number of red and blue points (ligand zone with $\rho_{L}>0.001$ au) is N_points_ = 430,976.

All other proposed complementarity factors CF2 and CF3 also demonstrate good correlation with SUMRLRE descriptor. Squared correlation coefficients of the dependencies (12) of CF2 and CF3 factors (R2(CF2) and R2(CF3)) in most cases are a little smaller than R2(CF1). Figure 1 demonstrates the dependencies on the example of 4kfj complex (red points).

Good correlations are observed for both cases, namely for the zones of intermolecular contacts (red points in Figure 1) and for the ligand zone (red and blue points in Figure 1). Parameters and statistical characteristics of the relationships (4) for complexes without hydrogens are given in Table S1 of Supplementary Materials.

### 3.2. Complexes with Hydrogens

First of all, it was interesting to estimate the correctness of the “enzyme–ligand” complexes with added hydrogens. It is obvious that in the correct complexes, the inner orbitals of the ligand cannot overlap enzyme orbitals and vice versa. However, since the electron density functions tend to 0 at R_Am_→∞, but never reach value 0, then the overlaps can be small but different from 0.

Consideration of $\rho_{L\left(inner\right)}^{}$ and $\rho_{E\left(inner\right)}^{}$ values in the ligand zone showed the “enzyme inner shells–ligand inner shells” overlaps tend to zero, i.e., $n_{L\left(inner\right)\cap E\left(inner\right)}<1.0\times 10^{-10}\;e$, though, the outer orbital of the receptor may penetrate slightly into the inner orbital of the ligand and vice versa. The number of electrons in the overlaps of the inner and outer orbitals of the receptor and ligands ($n_{L\left(inner\right)\cap E\left(outer\right)}$ and $n_{L\left(outer\right)\cap E\left(inner\right)}$) are insignificant. Only in the case of 4kfj, the overlap $n_{L\left(inner\right)\cap E\left(outer\right)}$ = 0.0026 e is perceptible, in other cases $n_{L\left(inner\right)\cap E\left(outer\right)}$ and $n_{L\left(outer\right)\cap E\left(inner\right)}$ equal 3$.0\times 10^{-9}-3.0\times 10^{-4}\;e$. In most cases they refer to the overlap between the hydrogen atom and the inner orbital of the electronegative atom (N or O).

In the 4kfj complex, the $n_{L\left(inner\right)\cap E\left(outer\right)}=0.0026\;e$ is provided by the H(H2O)…N(ligand) contact whose distance equals 1.73 Å. This is a typical hydrogen bond which does not depend on the tautomerism. However, we must bear in mind that AlteQ was developed for reproducing the electron density registered using low temperature X-ray diffraction at the temperature of liquid nitrogen (−196 °C). The vibration of hydrogens is very significant at temperatures greater than the room temperature, and the electron density of the hydrogens can be smeared in the intermolecular space of hydrogen bonds reducing $n_{\left(inner\right)\cap \left(outer\right)}$ in the real organism. Thus, the distribution of inner orbitals of all considered tautomers does not contradict the generally accepted concepts and $n_{\left(inner\right)\cap \left(outer\right)}$ may have insignificant value for typical hydrogen bonds.

We tried to consider less stable tautomeric forms of the complexes and found that their $n_{\left(inner\right)\cap \left(outer\right)}$ value increased. For example, the most stable tautomer of the 1hfp complex has $n_{inner\cap outer\;}=4.72\times 10^{-5}\;e$, while one of the least stable tautomers of the 1hfp complex has 22.69 times greater value, i.e., $n_{inner\cap outer}\;$= 0.001071 e, and relate to the H(COO of the ligand)…N(enzyme) overlap. At that, the maximum value of the overlap function of the inner and outer orbitals is $\rho_{L\left(inner\right)\cap E}=4.206\times 10^{-6}e$ for the most stable modeled tautomer of the complex while for the less stable tautomers $\rho_{L\cap E\left(inner\right)}=$ 0.0001564 e and $\rho_{L\cap E\left(inner\right)\;}$= 0.001223 e, respectively (Table 2).

**Figure 2:**
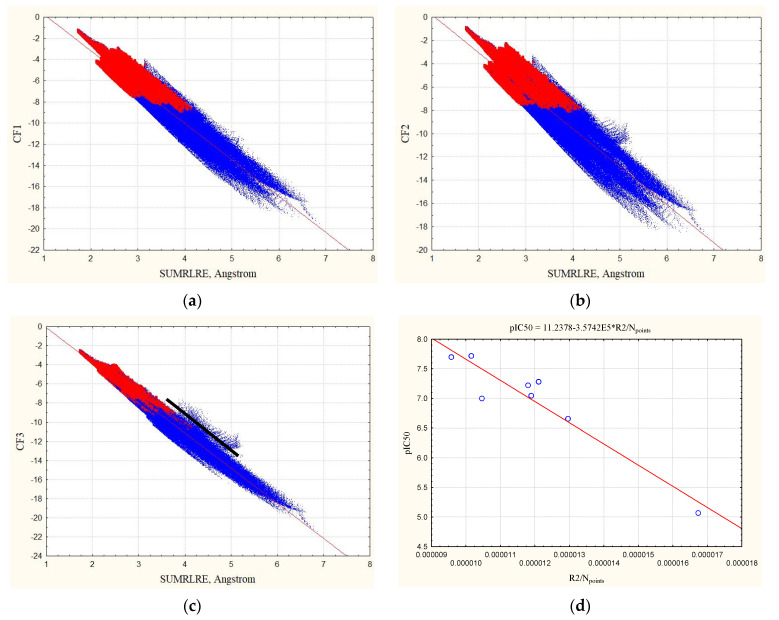
The dependencies of the CF1 (**a**), CF2 (**b**), CF3 (**c**) complementarity factors with SUMRLRE on the example of 4kfj complex with hydrogens. Red points (N_points_ = 80045) show the dependencies for the zones of intermolecular contacts, red and blue points show the dependencies for the ligand zone (the number of points N_points_ = 528,073). (**d**) The dependency of pIC50 values of ligands with R2/N_points_ obtained for the Equation (12) and CF3 factor.

The examples of the dependencies of the complementarity factors with SUMRLRE are shown in Figure 2 for the 4kfj complex, squared correlation coefficient (R2), standard error of the estimate (Sigma), and maximum values of the factors (maxCF) are given in the Table S1 of Supplementary Materials, both for the zones of intermolecular contacts (with $\rho_{E}>0.001$ au and $\rho_{L}>0.001$ au) and for the ligand zone (with $\rho_{L}>0.001$ au, red points).

You can see that hydrogens degrade the quality of the dependencies (12) for both the published complementarity factor CF1 and the factor CF2 when the zones of intermolecular contacts (red dots in Figure 2 with $\rho_{E}>0.001$ au and $\rho_{L}>0.001$ au) are considered in comparison with the ligand zone, and in comparison with the complexes without hydrogens. The quality of the dependency (12) for CF3 factor remains high, both for the zones of intermolecular contacts and for the ligand zone. Furthermore, Table S2 shows that a- and b-parameters of the CF3 = f(SUMRLRE) relationships differ insignificantly, a-parameters differ from each other on 0.4–6.4% while b-parameters on 0.07–2.74%. The minimal differences are observed for 3gi2 complex (Equations (21)–(24)):

(21) $$\Delta a_{CF3},\%=\frac{2\times \left(3.4793-3.4937\right)\times 100\%}{\left(3.4793+3.4937\right)}=0.4\%,$$

(22) $$\Delta b_{CF3},\%=\frac{2\times \left|-3.6474+3.6501\right|\times 100\%}{\left(-3.6474-3.6501\right)}=0.07\%,$$

The maximal differences are observed for 3ntz complex:

(23) $$\Delta a_{CF3},\%=\frac{2\times \left(3.8201-3.5825\right)\times 100\%}{\left(3.8201+3.5825\right)}=6.4\%,$$

(24) $$\Delta b_{CF3},\%=\frac{2\times \left|-3.7676+3.6657\right|\times 100\%}{\left(-3.7676-3.6657\right)}=2.74\%,$$

Consequently, the CF3 factor looks more universal; it works well when considering the structures of complexes with hydrogen atoms both in the zones of intermolecular contacts and in the ligand zone. Statistical characteristics (R2, Sigma) of the dependencies (12) CF3 = f(SUMRLRE) can be used as the criteria of complementarity of complexes with hydrogens, even for the zones of intermolecular contacts.

**Figure 3:**
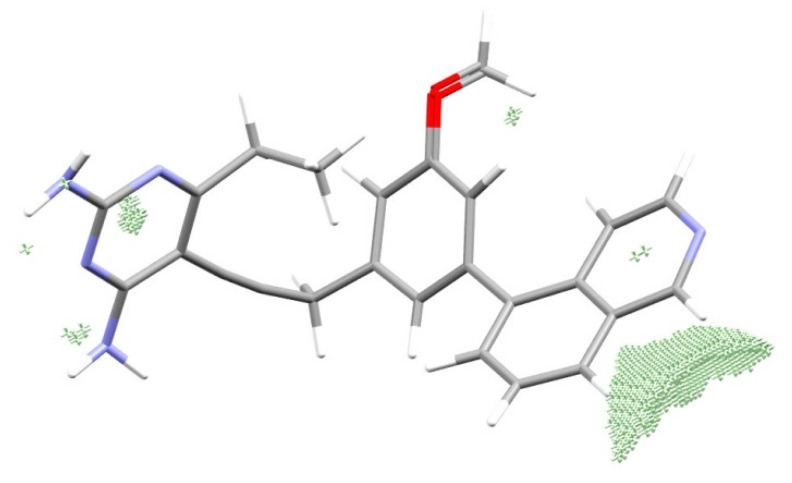
Space localization of the points with the maximum deviation of the predicted (Equation (12)) and observed CF3 (Equation (16)) factors.

The points with the maximum deviation of the predicted and observed CF3 factors CF3 (observed, Equation (16))–CF3(predicted using Equation (12)), and laying above the black line in Figure 1c,d and 2d, are located in the zones of the ligand’s molecular space shown in Figure 3. These are points that are almost at the same distance from several atoms, so there are several atoms contributing almost equally to the electron density at the points.

**Figure 4:**
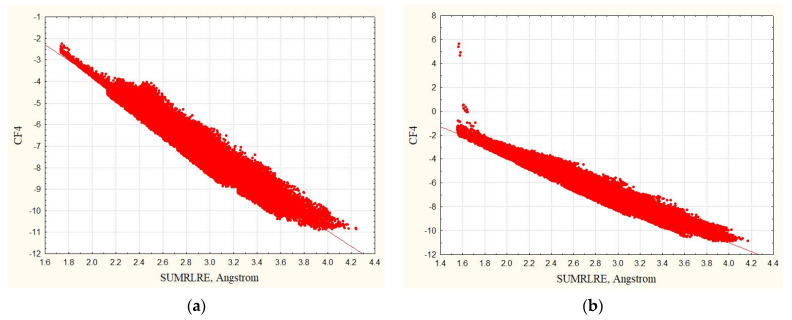
The dependencies of *CF4* complementarity factor with SUMRLRE: (**a**) 4kfj complex with hydrogens, (**b**) modified and incorrect complex 4kfj with hydrogens and the ligand displaced by 0.45 Å (the minimum distance between contacting atoms is 1.55 Å). Red points (N_points_ = 80,045) show the dependencies for the zones of intermolecular contacts.

Since for all considered complexes the overlaps of inner orbitals are insignificant, then CF3≈CF4. However, CF4 factor is sensitive to the incorrect ligand pose with the pronounced overlaps of inner orbitals. This can be demonstrated by the example of complex 4kfj. We modified it by displacing the ligand in the receptor cavity by 0.45 angstroms so that the minimum distance between the contacting atoms becomes 1.55 Å. Figure 4 demonstrates how much the quality of dependence CF4 = f(SUMRLRE) for the modified analogue of 4kfj complex has deteriorated.

### 3.3. The Relationship of Experimental pIC50 Values with Statistical Characteristics of Equation (12)

We collected data on the values of inhibitory concentrations (IC50) of eight ligands out the nine ligands presented in this article from the Binding Database in order to demonstrate experimental control. We found the negative decimal logarithm of the IC50 values; they are given in the Table S3 of the Supplementary Materials. The pIC50 values of the ligands characterize the reactivity of the ligands with respect to DHFR. The dependence of the pIC on the R2/N_points_ values (namely, the squared correlation coefficient, divided by the number of points in the overlap zone obtained for the Equation (12) and CF3 factor) was found. The relationship is presented in Figure 2d, and its correlation coefficient equals 0.95. So, these characteristics assessing the complementarity can be used for prognosis of bioactivity of compounds.

### 3.4. Reconstruction of the Pattern of the Electronic Structure of the Drug Suitable for the Given Enzyme

Equations (12) and (14) can be transformed (Equation (25)).

(25) $$\left\{\left(\frac{\rho_{L}\cdot \rho_{l\left(CNT\right)}}{N_{l}}\right)exp\left(-b_{CF1}\cdot R_{ml}\right)\right\}\left\{\left(\frac{\rho_{E}\cdot \rho_{e\left(CNT\right)}}{N_{e}}\right)exp\left(-b_{CF1}\cdot R_{me}\right)\right\}=exp\left(a_{CF1}\right),$$

Then, designating (Equations (26) and (27))

(26) $$\sigma_{L\left(CF1\right)}^{}=\left(\frac{\rho_{L}\cdot \rho_{l\left(CNT\right)}}{N_{l}}\right)exp\left(-b_{\mathrm{CF}1}\cdot R_{ml}\right),$$

(27) $$\sigma_{E\left(CF1\right)}^{}=\left(\frac{\rho_{E}\cdot \rho_{e\left(CNT\right)}}{N_{e}}\right)exp\left(-b_{\mathrm{CF}1}\cdot R_{me}\right),$$

we can obtain (Equation (28)):

(28) $$\sigma_{E\left(CF1\right)}\cdot \sigma_{L\left(CF1\right)}=exp\left(a_{CF1}\right),$$

Analogously, based on the Equations (12), (15)–(17) we can determine $\sigma_{L\left(CF2\right)}^{}$ and $\sigma_{E\left(CF2\right)}^{}$ values (Equations (29) and (30)):

(29) $$\sigma_{L\left(CF2\right)}^{}=\left(\frac{\rho_{L}\cdot \rho_{l\left(CNT\right)}}{N_{l\left(outer\right)}}\right)exp\left(-b_{CF2}\cdot R_{ml}\right),$$

(30) $$\sigma_{E\left(CF2\right)}^{}=\left(\frac{\rho_{E}\cdot \rho_{e\left(CNT\right)}}{N_{e\left(outer\right)}}\right)exp\left(-b_{CF2}\cdot R_{me}\right)$$

and $\sigma_{L\left(CF3\right)}^{}$ and $\sigma_{E\left(CF3\right)}^{}$ values (Equations (31) and (32)):

(31) $$\sigma_{L\left(CF3\right)}^{}=\left(\frac{\rho_{L}\cdot \rho_{l\left(CNT\right)}}{N_{l\left(outer\right)}^{2}}\right)exp\left(-b_{CF3}\cdot R_{ml}\right),$$

(32) $$\sigma_{E\left(CF3\right)}^{}=\left(\frac{\rho_{E}\cdot \rho_{e\left(CNT\right)}}{N_{e\left(outer\right)}^{2}{}_{}}\right)exp\left(-b_{CF3}\cdot R_{me}\right)$$

and $\sigma_{L\left(CF4\right)}^{}$ and $\sigma_{E\left(CF4\right)}^{}$ values (Equations (33) and (34)):

(33) $$\sigma_{L\left(CF4\right)}^{}=\left(\frac{\rho_{L}\cdot \rho_{l\left(CNT\right)}}{N_{l\left(outer\right)}^{2}}\right)exp\left(\rho_{L\left(inner\right)}\right)exp\left(-b_{CF4}\cdot R_{ml}\right),$$

(34) $$\sigma_{E\left(CF4\right)}^{}=\left(\frac{\rho_{E}\cdot \rho_{e\left(CNT\right)}}{N_{e\left(outer\right)}^{2}{}_{}}\right)exp\left(\rho_{E\left(inner\right)}\right)exp\left(-b_{CF4}\cdot R_{me}\right)$$

*a_CFk_* and *b_CFk_*-parameters that have been determined for the most effective ligands can be used to build a pattern of the electronic structure of the desired drug. This image can be presented in general form as follows (Equation (35)):

(35) $$\sigma_{L\left(CFk\right)}=\exp\left(a_{CFk}\right)/\sigma_{E\left(CFk\right)},$$

Further, a new molecule can be superimposed on the given pattern until their maximum coincidence. In this case, we will be able to determine the most complementary position of the new ligand in the receptor cavity.

### 3.5. A Way towards Analytical Docking Procedure

Based on the dependency (12) and Equations (14)–(16) and knowing parameters of the Equation (12) determined for a concrete receptor, we can suggest an approach for an analytical solution of the problem of molecular docking.

Consider the mth points that are located in the middle of the intermolecular contact of eth enzyme’s atom and lth ligand’s atom. Then the distance $R_{ml}=R_{me}=\frac{1}{2}R_{le}$.

In the case when the mth point is closest to the atoms e and l and the contribution of neighboring atoms to the electron density at the mth point is much lower than the contributions of the atoms e and l, then for elements of 1–3 periods, including organogens, it is possible to write (Equations (36) and (37)):

(36) $$\rho_{E}\approx \rho_{em\left(outer\right)}=a_{e_{n_{e}sp}}\exp\left(-b_{e_{n_{e}sp}}R_{me}\right),$$

(37) $$\rho_{L}\approx \rho_{lm\left(outer\right)}=a_{l_{n_{l}sp}}\exp\left(-b_{l_{n_{l}sp}}R_{ml}\right)$$

Then we can determine distances of the most effective contacts between atoms l and e in the “enzyme–ligand” complex based on the $a_{CFk}$ and $b_{CFk}$ parameters and Equations (14)–(16):

(38) $$R_{le}=2\frac{ln\frac{a_{e_{n_{e}sp}}^{2}}{N_{e}^{}}+ln\frac{a_{l_{n_{l}sp}}^{2}}{N_{l}^{}}-a_{CF1}}{2\cdot b_{CF1}-b_{e_{n_{e}sp}}-b_{l_{n_{l}sp}}},$$

(39) $$R_{le}=2\frac{ln\frac{a_{e_{n_{e}sp}}^{2}}{N_{e\left(outer\right)}^{}}+ln\frac{a_{l_{n_{l}sp}}^{2}}{N_{l\left(outer\right)}^{}}-a_{CF2}}{2\cdot b_{CF2}-b_{e_{n_{e}sp}}-b_{l_{n_{l}sp}}}$$

(40) $$R_{le}=2\frac{ln\frac{a_{e_{n_{e}sp}}^{2}}{N_{e\left(outer\right)}^{2}}+ln\frac{a_{l_{n_{l}sp}}^{2}}{N_{l\left(outer\right)}^{2}}-a_{CF3}}{2\cdot b_{CF3}-b_{e_{n_{e}sp}}-b_{l_{n_{l}sp}}}$$

In the case of correct non-covalently bound complexes, both $\rho_{E\left(inner\right)}$ and $\rho_{L\left(inner\right)}$ tend to 0 and CF3 = CF4 for the zones of intermolecular contacts, therefore Equation (13) is suitable for CF3 and CF4 factors.

High correlation coefficients of dependences (12) show that in the formation of a complex, the most effective are intermolecular contacts which provide predominantly paired atom–atomic interactions, while interactions of the bifurcate and more disoriented type are minimized, in which the contributions of neighbors to the mth point become comparable to the contributions of atoms e and l. Those interactions are carried out along lines (we will call them contact lines) providing the maximum contribution of e and l atoms and the minimum contribution of neighbors covalently bound to e and l atoms.

**Figure 5:**
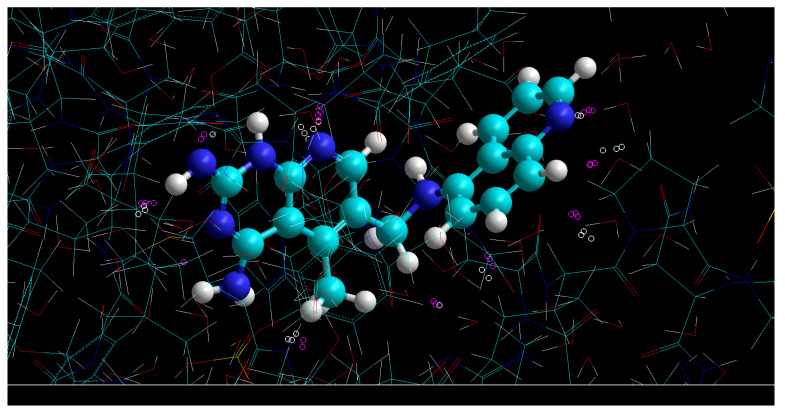
The most effective contacts of the ligand and the enzyme in the 1kms complex, predicted contact areas are shown by violet (ligand’s areas) and white (enzyme’s areas) points.

These regions of space are very close to the lines along which the maximum distance from atoms–neighbors is observed. This statement is in good agreement with the theory of Ronald Gillespie and Ronald Nyholm [34,35] that overlaps are carried out along lines that ensure the maximum distance of electron pairs, and hence ligands of the central atom (in this case, a ligand is an atom or a group of atoms connected to the central atom) from each other. The contact areas of the enzyme and the ligand are in contact with each other, shown in Figure 5 on the example of 1kms complex.

**Figure 6:**
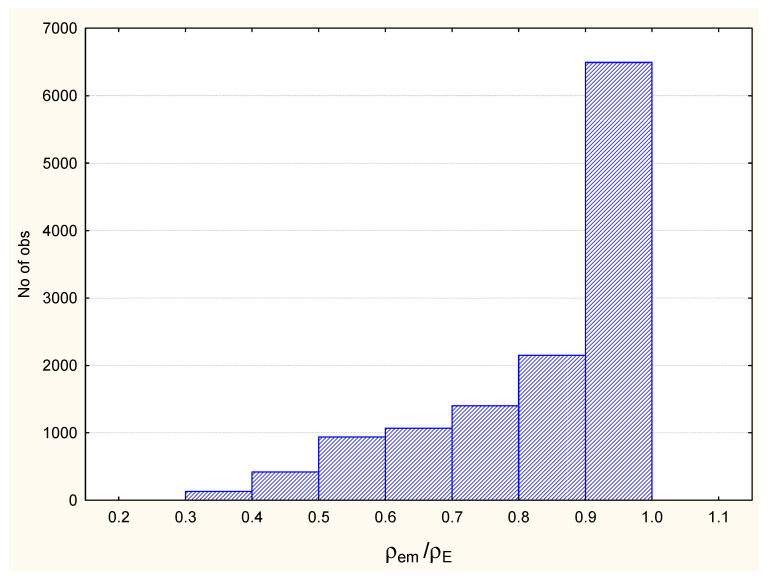
The number of points in different ranges of $\rho_{em}/\rho_{E}$ values for 1kms complex.

On the example of the 1kms complex, Figure 6 demonstrates that 10,047 out of 12,607 (80%) points of the zones of intermolecular contacts have contribution of one atom to the electron density on the level of 70–100% (e.g., $\frac{\rho_{em}}{\rho_{E}}\geq 0.7$).

Basically, these are the points responsible for the formation of the most effective interactions—hydrogen bonds O-H…O, N-H…O, etc. The contribution of neighbors in intermolecular interactions is noticeable in pi-stacking (C…C, C…N intermolecular contacts) and in C…H contacts, therefore the Equations (38)–(40) should give incorrect values for such intermolecular interactions and can only work for predicting the lengths of covalent C-C and C-H bonds, when at small distances from the nucleus (for example, the middle of a covalent bond) the electron density increases many times in comparison with the electron density of neighbors due to the exponential function of the electron density.

Distances of the most effective contacts determined using Equations (38) and (40) and $a_{CF1}$, $b_{CF1}$, $a_{CF3}$, $b_{CF3}$ parameters of the complementarity factors *CF1*, *CF3,* and *CF4* are given in Table 3 and Table 4 on the example of 1kms complex. Table 5 demonstrates observed distances in the 1kms complex.

Hydrogen atoms are at the periphery of the molecule, in general, many N…N, N…O, O…O contacts are made through hydrogen atoms, i.e., they are N-H…N, N-H…O, N…H-O, O-H…O hydrogen bonds. The CF3 and CF4 factors take into account the presence of hydrogens, so the lengths of direct N…N, N…O, O…O contacts are best calculated from CF1 factor for the complex without hydrogens in the zone of intermolecular interactions. Thus, these CF1, CF3, CF4 factors are additional to each other. The obtained distances (Table 3 and 4) can be realized only in this enzyme, apparently, in the conformation of the considered 1kms complex. Obviously, not all molecules are capable of matching the enzyme pocket. Some distances are too low and cannot be realized in the non-covalently bound complex (direct N…N, N…O, O…O contacts with the distances 1.85, 2.02, 2.13 Å (Table 4), respectively), because there is an overlap of the inner orbitals, uncharacteristic for intermolecular interactions, see Section 3.1 and Section 3.2), which will lead to a deterioration of the correlation coefficient for the CF4 factor. Therefore, the distances that can or should be realized to ensure effective binding to the receptor are highlighted in Table 3 and Table 4. You can see most of the most effective contacts are observed in the 1kms complex. Table 5 shows that in the 1kms complex there are typical N-H…O, N…H-O hydrogen bonds, which are very effective and have typical distances >2.76 Å between the electronegative atoms (predicted value is 2.88 Å). Observed N…N contacts are characterized by rather long distances >3.52 Å and cannot be referred to the effective hydrogen bonds. Apparently, these N…N contacts are forced, arising as a result of other effective interactions. However, predicted value 2.75 Å shows that for another ligand, N…N type of interactions trough hydrogen atom (N…H-N) is possible in this receptor pocket. Predicted values of distances 1.75 Å and 1.97 Å for H…N and H…O are close to the observed values—1.83 and 1.90 Å.

Therefore, analytical docking of a new ligand can be based on the estimation of preferred lengths of the most effective intermolecular contacts typical for the enzyme. Then the docking procedure is reduced to the following steps:

(1) Computation of the distances (Equations (38)–(40)) of the most effective contacts and the most preferred for a given enzyme.
(2) The construction of contact zones for the ligand and the enzyme; these zones determine the direction of intermolecular interactions between the ligand and the enzyme.
  In order to find these zones, you need to find coordinates of points in the basin of eth atom ($\Omega_{e}$), which will satisfy the conditions:
  (41) $$\frac{\partial }{\partial x_{\Omega_{e}}}\sum_{\begin{array}{c} i\in enzyme\\ i=1\\ i\neq e \end{array}}^{N_{e}}\rho_{i}=0,$$
  (42) $$\frac{\partial }{\partial y_{\Omega_{e}}}\overset{N_{e}}{\underset{\begin{array}{c} i\in enzyme\\ i=1\\ i\neq e \end{array}}{\sum }}\rho_{i}=0$$
  (43) $$\frac{\partial }{\partial z_{\Omega_{e}}}\overset{N_{e}}{\underset{\begin{array}{c} i\in enzyme\\ i=1\\ i\neq e \end{array}}{\sum }}\rho_{i}=0$$
  and will be the minimum points of the electron density function of contributions of neighbors of the *e*th atom.
(3) In order to find the contact zones in the ligand, the same can be performed for the periphery ligand atoms.
(4) The correct binding position of the ligand can then be determined from the condition of maximum overlap between the contact zones of the enzyme and the ligand.

The proposed docking algorithm based on the maps of the electron density of the ligands will make it possible to predict the structure of the receptor–ligand complexes. For the docked complexes of DHFR, having no overlaps of inner shells, the quality of the Equation (12) for CF3 or CF4 complementarity factors, namely R2/N_points_, can be estimated. After that, the Equation shown in Figure 2d will allow predicting pIC50 for DHFR inhibitors. A low predicted value will be a reason to exclude the inactive compound from further biological testing.

## 4. Conclusions

In this work, we suggested new approaches for assessment of enzyme–ligand complementarity. The approaches are based on the calculation of 3D maps of electron density of “receptor–ligand” complexes. The work of the complementarity factors was demonstrated on the complexes of human DHFR with ligands, taking into account hydrogens. For the first time, the possibility of overlapping inner orbitals was taken into account, which can later be used to discard incorrect docked structures. We found the relationship between experimental pIC50 values of ligands and the characteristic of the complementarity assessment, namely R2/N_points_. We found that formation of an enzyme–ligand complex is based on the formation of the most effective intermolecular contacts, which provide predominantly paired atom–atomic interactions, while interactions of the bifurcate and more disoriented type are minimized. Algorithm of analytical docking procedure based on the enzyme–ligand complementarity factors has been suggested.

## Figures and Tables

**Table 1:** The number of electrons in the “enzyme–ligand” overlaps ($n_{L\cap E}$) and the number of electrons donated by the ligand ($n_{L\cap E}^{ligand}$) to the overlaps.

Complexes	$n_{L\cap E},\;e$	$n_{L\cap E}^{ligand},\;e$
1boz	0.2131	0.1737
1hfp	0.3188	0.2494
1kms	0.3118	0.2465
3ghc	0.4099	0.3255
3gi2	0.4606	0.3623
3ntz	0.3673	0.2947
3nu0	0.3943	0.3162
4kfj	0.4430	0.3256
4qhv	0.3174	0.2387

**Table 2:** MM3-MERA force field energy, the shortest distance between enzyme and ligand contacting atoms (*R_el_*,), the maximum values of $\rho_{L\left(inner\right)\cap E}$ and $\rho_{L\cap E\left(inner\right)}$ overlap functions of different modeled tautomers of the 1hfp complex.

	1hfp (Tautomer 1)	1hfp (Tautomer 2)	1hfp (Tautomer 3)
MM3-MERA force field energy, kcal/mol	−2406.70678	−2398.00307	−2341.35311
*R_el_*, Å	1.9126	1.5514932	1.6928
$\rho_{L\left(inner\right)\cap E}$, e/Å^3^	0.000004206	0.000004206	0.00004766
$\rho_{L\cap E\left(inner\right)}$, e/Å^3^	0.000002932	0.000156418	0.001223

**Table 3:** The most effective contacts predicted using CF1 factor.

	$N_{e}^{}=7$	$N_{e}^{}=8$
$N_{l}^{}=7$	2.75 ^1^	2.88 ^1^
$N_{l}^{}=8$	2.88 ^1^	2.96 ^1^
$N_{l}^{}=9$	2.83 ^1^	2.90 ^1^

^1^ Distances that can correspond to intermolecular contacts, which are characterized by a small overlap of inner orbitals.

**Table 4:** The most effective contacts predicted using CF3 factor.

	$N_{e}^{}=1$	$N_{e}^{}=7$	$N_{e}^{}=8$
$N_{l}^{}=1$	1.63 ^1^	1.75 ^1^	1.97 ^1^
$N_{l}^{}=7$	1.75 ^1^	1.85	2.02
$N_{l}^{}=8$	1.97 ^1^	2.02	2.13

^1^ Distances that can correspond to intermolecular contacts, which are characterized by a small overlap of inner orbitals.

**Table 5:** The most effective contacts observed in the 1kms complex.

	$N_{e}^{}=1$	$N_{e}^{}=7$	$N_{e}^{}=8$
$N_{l}^{}=1$	1.94 ^1^–3.34	1.83 ^1^–3.66	1.90 ^1^–3.49
$N_{l}^{}=7$		3.52–3.91	2.76 ^1^–3.75
$N_{l}^{}=8$		2.76 ^1^–3.75	-

^1^ Distances that correspond to short intermolecular contacts and match the predicted value well.

## Data Availability

Console applications of the software for the AlteQ complementarity assessment (alteq_map_enzyme_ligand_4_win32.exe for Windows and alteq_map_enzyme_ligand_4_linux for Linux) are available at [36] free of charge.

## Supplementary Materials

The following are available online at https://www.mdpi.com/article/10.3390/life11090983/s1, Table S1: “*a*- and *b*- parameters, squared correlation coefficients (R2), standard error of estimate (Sigma), number of points (Npoints) of the dependencies (12) for CF1, CF2 and CF3 complementarity factors determined in the zones of intermolecular contacts (with $\rho_{E}>0.001$ au and $\rho_{L}>0.001$ au) and in the ligand zone (with $\rho_{L}>0.001$ au) for complexes without hydrogens”; Table S2: “Squared correlation coefficient (R2), standard error of the estimate (Sigma), maximal values (maxCF) of CF1, CF2, CF3 complementarity factors, *a*- and *b*-parameters of the Equations (14)–(16) for complexes with hydrogens.”
