# Supplementary material for: The Complementarity Principle—One More Step towards Analytical Docking on the Example of Dihydrofolate Reductase Complexes
Source: Life (Basel). 2021 Sep 19;11(9):983. doi: 10.3390/life11090983 (PMC8469765; doi:10.3390/life11090983)
Supplement: Supplementary file 1 [file life-11-00983-s001.zip › life-1392188-supplementary.pdf]

life

MDPI

Article

The Complementarity Principle—One More Step towards Analytical Docking on the Example of Dihydrofolate Reductase Complexes

Vladimir Potemkin *, Maria Grishina *

Laboratory of Computational Modelling of Drugs, South Ural State University, 454080 Chelyabinsk, Russia
* Correspondence: potemkinva@susu.ru (V.P.); grishinama@susu.ru (M.G.)

Supplementary materials

Table S1. *a*- and *b*- parameters, squared correlation coefficients (R2), standard error of estimate (Sigma), number of points (Npoints) of the dependencies (12) for CF1, CF2 and CF3 complementarity factors determined in the zones of intermolecular contacts (with $\rho_E > 0.001$ a.u. and $\rho_L > 0.001$ a.u.) and in the ligand zone (with $\rho_L > 0.001$ a.u.) for complexes without hydrogens.

		zones of intermolecular contacts				The ligand zone			
		a	b	R2	Sigma	a	b	R2	Sigma
1boz	CF1	5.0515	-3.5988	0.8763	0.3901	4.9217	-3.6335	0.9074	0.6732
	CF2	5.2442	-3.4449	0.8853	0.3579	5.4462	-3.5799	0.9015	0.6862
	CF3	0.3796	-2.9230	0.8747	0.3193	1.7106	-3.3997	0.8691	0.7652
	Npoints	7531				343105			
1hfp	CF1	6.4343	-3.9491	0.9513	0.2881	5.8690	-3.8324	0.9478	0.6396
	CF2	6.5653	-3.7843	0.9470	0.2887	6.3781	-3.7783	0.9439	0.6552
	CF3	1.4782	-3.2220	0.9055	0.3356	2.5821	-3.5946	0.9207	0.7500
	Npoints	12414				418670			
1kms	CF1	5.3104	-3.6297	0.9541	0.2388	4.4625	-3.4882	0.9373	0.5112
	CF2	5.2777	-3.4123	0.9487	0.2380	4.8976	-3.4103	0.9316	0.5236
	CF3	-0.3443	-2.6771	0.8854	0.2888	0.8583	-3.1474	0.8979	0.6015
	Npoints	12606				336873			
3ghc	CF1	6.2702	-3.9250	0.9462	0.3103	5.5578	-3.7759	0.9249	0.6655
	CF2	5.2747	-3.3954	0.9323	0.3032	6.0782	-3.7049	0.9228	0.6624
	CF3	0.0662	-2.8138	0.8867	0.3333	2.1147	-3.4850	0.8889	0.7617
	Npoints	16398				452695			
3gi2	CF1	6.2361	-3.9198	0.9462	0.3074	5.0877	-3.6479	0.9232	0.5965
	CF2	5.2838	-3.4014	0.9323	0.3014	5.4557	-3.5387	0.9250	0.5715
	CF3	0.1414	-2.8396	0.8849	0.3369	1.2559	-3.2601	0.8916	0.6445
	Npoints	18925				436741			
3ntz	CF1	5.8369	-3.8037	0.9271	0.3110	5.3645	-3.7168	0.9315	0.6765
	CF2	4.5652	-3.1928	0.9161	0.2818	6.0983	-3.7047	0.9281	0.6920
	CF3	-0.4610	-2.6594	0.8723	0.2966	2.4020	-3.5440	0.8990	0.7973
	Npoints	14983				468184			
3nu0	CF1	6.3647	-3.9617	0.9500	0.2905	5.2029	-3.6938	0.9147	0.7098
	CF2	5.2090	-3.3818	0.9365	0.2815	5.8742	-3.6684	0.9079	0.7349
	CF3	0.2509	-2.8741	0.9035	0.3002	2.0621	-3.4820	0.8667	0.8591
	Npoints	15595				480706			
4kfj	CF1	5.5122	-3.6939	0.9123	0.3145	5.1904	-3.6895	0.9517	0.5722
	CF2	5.8686	-3.5780	0.9117	0.3058	5.6936	-3.6223	0.9447	0.6038
	CF3	1.5949	-3.1957	0.8461	0.3743	2.1728	-3.4754	0.9211	0.7008
	Npoints	20129				430975			
4qhv	CF1	5.1875	-3.5992	0.9514	0.2906	4.5604	-3.5238	0.9306	0.5633
	CF2	5.5656	-3.4991	0.9497	0.2875	5.0826	-3.4660	0.9262	0.5727
	CF3	1.2950	-3.1494	0.9178	0.3367	1.3370	-3.2712	0.9000	0.6384
	Npoints	12787				320736			

Table S2. Squared correlation coefficient (R2), standard error of the estimate (Sigma), maximal values (maxCF) of CF1, CF2, CF3 complementarity factors, *a*- and *b*-parameters of the eqs. (14)-(16) for complexes with hydrogens.

name		zones of intermolecular contacts				the ligand zone			
		a	*b*	R2	Sigma	*a*	*b*	R2	Sigma
1boz	CF1	2.0491	-2.7789	0.7924	0.5833	3.1855	-3.3033	0.8872	0.7693
	CF2	1.3253	-2.4286	0.6493	0.7320	2.8139	-3.1026	0.8243	0.9356
	CF3	3.7025	-3.6901	0.9548	0.3292	3.7431	-3.7490	0.9600	0.4997
	Npoints	57046				425086			
1hfp	CF1	3.1851	-3.1347	0.8070	0.6016	4.1803	-3.5511	0.9255	0.7851
	CF2	2.7512	-2.8760	0.7022	0.7350	4.0433	-3.4132	0.8885	0.9424
	CF3	3.6636	-3.6902	0.9385	0.3707	3.8843	-3.7838	0.9719	0.5010
	Npoints	72436				505187			
1kms	CF1	2.7818	-3.0011	0.8414	0.5675	3.0402	-3.1793	0.9140	0.6634
	CF2	2.2445	-2.7089	0.7303	0.7169	2.6652	-2.9603	0.8561	0.8259
	CF3	3.4784	-3.6066	0.9612	0.3154	3.3369	-3.5826	0.9714	0.4185
	Npoints	71166				405154			
3ghc	CF1	3.5934	-3.2958	0.8516	0.6156	3.7625	-3.4268	0.8923	0.7712
	CF2	2.7546	-2.8647	0.7604	0.7195	3.4974	-3.2268	0.8402	0.9115
	CF3	3.7244	-3.7231	0.9534	0.3684	3.6796	-3.7204	0.9603	0.4901
	Npoints	93878				532127			
3gi2	CF1	3.3654	-3.2029	0.8462	0.5825	3.5873	-3.3431	0.8993	0.7044
	CF2	2.5859	-2.7935	0.7492	0.6895	3.2013	-3.0995	0.8525	0.8115
	CF3	3.4937	-3.6501	0.9480	0.3646	3.4793	-3.6474	0.9605	0.4653
	Npoints	98969				509414			
3ntz	CF1	2.3359	-2.8732	0.8213	0.5817	3.5878	-3.3905	0.9144	0.8178
	CF2	1.2313	-2.3615	0.6757	0.7101	3.3839	-3.2207	0.8660	0.9990
	CF3	3.5825	-3.6657	0.9536	0.3510	3.8201	-3.7676	0.9706	0.5168
	Npoints	80161				547510			
3nu0	CF1	2.9333	-3.0580	0.8235	0.5812	3.5702	-3.3755	0.9086	0.7674
	CF2	1.9739	-2.5978	0.6932	0.7096	3.3200	-3.1911	0.8578	0.9315
	CF3	3.6475	-3.6784	0.9463	0.3599	3.8003	-3.7571	0.9632	0.5267
	Npoints	89037				562141			
4kfj	CF1	2.1146	-2.7442	0.7431	0.6200	3.6902	-3.4337	0.9105	0.7775
	CF2	1.4593	-2.3955	0.5765	0.7889	3.4566	-3.2626	0.8571	0.9623
	CF3	3.4639	-3.6018	0.9444	0.3358	3.5617	-3.6757	0.9680	0.4827
	Npoints	80045				528073			
4qhv	CF1	2.6660	-2.9614	0.7650	0.6333	3.4879	-3.3546	0.8964	0.7177
	CF2	2.1401	-2.6716	0.6355	0.7806	3.2569	-3.1860	0.8402	0.8747
	CF3	3.4287	-3.5815	0.9414	0.3449	3.5780	-3.6749	0.9586	0.4806
	Npoints	77735				404355			

Table S3. The negative decimal logarithm of the inhibitory concentrations (pIC50) of 8 ligands [23-29].

ligand	pIC50 (IC50 is measured in mol/l)
1boz	5.07
1hfp	6.66
1kms	No found IC50 values for human DHFR
3ghc	7.72
3gi2	7.70
3ntz	7.05
3nu0	7.00
4kfj	7.22
4qhv	7.28
